# Precise execution of personalized surgical planning using three-dimensional printed guide template in severe and complex adult spinal deformity patients requiring three-column osteotomy: a retrospective, comparative matched-cohort study

**DOI:** 10.1186/s13018-024-04712-0

**Published:** 2024-05-04

**Authors:** Yangpu Zhang, Honghao Yang, Chaofan Han, Yiqi Zhang, Lijin Zhou, Yong Hai

**Affiliations:** Department of Orthopedic Surgery, Beijing Chao-Yang Hospital, Capital Medical University, Gongti South Rd, No. 8, Beijing, 100020 China

## Abstract

**Background:**

The surgical treatment of severe and complex adult spinal deformity (ASD) commonly required three-column osteotomy (3-CO), which was technically demanding with high risk of neurological deficit. Personalized three dimensional (3D)-printed guide template based on preoperative planning has been gradually applied in 3-CO procedure. The purpose of this study was to compare the efficacy, safety, and precision of 3D-printed osteotomy guide template and free-hand technique in the treatment of severe and complex ASD patients requiring 3-CO.

**Methods:**

This was a single-centre retrospective comparative cohort study of patients with severe and complex ASD (Cobb angle of scoliosis > 80° with flexibility < 25% or focal kyphosis > 90°) who underwent posterior spinal fusion and 3-CO between January 2020 to January 2023, with a minimum 12 months follow-up. Personalized computer-assisted three-dimensional osteotomy simulation was performed for all recruited patients, who were further divided into template and non-template groups based on the application of 3D-printed osteotomy guide template according to the surgical planning. Patients in the two groups were age- and gender- propensity-matched. The radiographic parameters, postoperative neurological deficit, and precision of osteotomy execution were compared between groups.

**Results:**

A total of 40 patients (age 36.53 ± 11.98 years) were retrospectively recruited, with 20 patients in each group. The preoperative focal kyphosis (FK) was 92.72° ± 36.77° in the template group and 93.47° ± 33.91° in the non-template group, with a main curve Cobb angle of 63.35° (15.00°, 92.25°) and 64.00° (20.25°, 99.20°), respectively. Following the correction surgery, there were no significant differences in postoperative FK, postoperative main curve Cobb angle, correction rate of FK (54.20% vs. 51.94%, *P* = 0.738), and correction rate of main curve Cobb angle (72.41% vs. 61.33%, *P* = 0.101) between the groups. However, the match ratio of execution to simulation osteotomy angle was significantly greater in the template group than the non-template group (coronal: 89.90% vs. 74.50%, *P* < 0.001; sagittal: 90.45% vs. 80.35%, *P* < 0.001). The operating time (ORT) was significantly shorter (359.25 ± 57.79 min vs. 398.90 ± 59.48 min, *P* = 0.039) and the incidence of postoperative neurological deficit (5.0% vs. 35.0%, *P* = 0.018) was significantly lower in the template group than the non-template group.

**Conclusion:**

Performing 3-CO with the assistance of personalized 3D-printed guide template could increase the precision of execution, decrease the risk of postoperative neurological deficit, and shorten the ORT in the correction surgery for severe and complex ASD. The personalized osteotomy guide had the advantages of 3D insight of the case-specific anatomy, identification of osteotomy location, and translation of the surgical planning or simulation to the real surgical site.

## Introduction

Severe and complex adult spinal deformity (ASD) presented characteristics of abnormal anatomical structures, rigidty of spinal flexibility, pronounced kyphoscoliosis, and with or without neurological impairment [1]. For such cases, timely surgical correction is necessary. Due to the severity and rigidity of the spinal deformity, posterior three-column osteotomy (3-CO), such as pedicle subtraction osteotomy (PSO) and vertebral column resection (VCR), was commonly required to correct the deformity [2, 3]. However, the 3-CO was technically demanding and the risk of neurological complications caused by this procedure was still high when performing by experienced surgeons. The previous studies reported that the incidence of postoperative neurological deficit by 3-CO was ranged from 4 to 23%, which significantly impacted the patients’ health-related quality of life [4–8]. As the improper closure of the osteotomy gap was main cause of unsatisfactory outcomes, several strategies have been tried to solve this problem. In our previous studies, we promoted a strategy to obtain optimal osteotomy planning through personalized computer-assisted three-dimensional simulation; however, the match between execution and simulation was not ideal enough [9]. Therefore, how to translate the planning to practice was still challenging.

With the advances in computer-assisted surgery and three-dimensional (3D) printing technology, personalized 3D-printed guide template based on preoperative planning has been gradually applied in spinal surgery [10, 11]. Through reverse engineering, patient-specific guide template could be designed according to the result of surgery simulation; then the guide template could be manufactured by 3D-printing using layer wise materials. Therefore, the surgical planning was translated into real surgical tools to assist surgeons in operation. In severe and complex spinal deformity surgery, the most common application of this strategy was in pedicle screw placement [12–14]. Our previous meta analysis revealed that using 3D-printed guide template could increase the accuracy of pedicle screw insertion compared with the free-hand technique in spinal deformity patients [15]. For more skill-demanding osteotomy procedure, an in-vitro study by Xin et al. revealed that the 3D-printed osteotomy guide template was feasible in 3-CO and a high precision was obtained [16]. In clinical settings, we reported the first case serials of severe and complex ASD patients who underwent 3-CO with the assistance of personalized 3D-printed guide template [1]. The diagnosis of these patients included adult idiopathic scoliosis, congenital scoliosis, kyphosis secondary to tuberculosis, and ankylosing spondylitis (AS). This study further verified the benefits of this technique. However, all previous studies were single-arm design [12, 17, 18]. The superiority of 3D-printed guide template over the free-hand technique in 3-CO procedures was still unknown.

The purpose of this study was to compare the efficacy, safety, and precision of 3D-printed osteotomy guide template and free-hand technique in the treatment of severe and complex ASD patients requiring 3-CO, through a comparative matched-cohort study design.

## Materials and methods

### Patient cohort

This was a single-centre retrospective comparative cohort study of patients with severe and complex ASD who underwent posterior spinal fusion and 3-CO between January 2020 to January 2023. All surgeries for the cases were performed by the same surgeon with 20 years of experience in treating complex spinal deformities. The inclusion criteria were as follows: (1) patients with scoliosis, kyphosis, or kyphoscoliosis undergoing posterior spinal fusion surgery; (2) the major curve Cobb angle > 80° in coronal plane and/or sagittal plane; (3) the flexibility of spinal deformity ≤ 25% or concomitant congenital/iatrogenic spinal fusion requiring 3-CO; and (4) etiology of congenital, tubercular, traumatic, iatrogenic or ankylosing spondylitis. All patients were followed up for a minimum of 12 months. The exclusive criteria were: (1) patients younger than 18 years old; (2) patients who could not tolerate the surgery; (3) patients who only undergo posterior column osteotomy; and (4) follow-up data was incomplete. Personalized computer-assisted three-dimensional osteotomy simulation was performed for all recruited patients; additional 3D-printed guide template for osteotomy based on the surgical planning were used for patients who were admitted to hospital after November 2021. Therefore, this cohort was divided into template group and non-template group, and the application of 3D-printed guide template was not randomized. This study was approved by the Research Ethics Committee of Beijing Chao-Yang Hospital and informed consent was taken from patients.

### Surgical simulation and execution

#### Three-dimensional spine model reconstruction

Patients’ CT scan data of the whole spine were collected with Digital Imaging and Communications in Medicine (DICOM) format (DICOM format data from Siemens CT machine, SOMATOM Sensation 16, Siemens AG, Forchheim, Germany). All the tomographic pictures were imported into Mimics Medical 21.0 (Materialise NV, Leuven, Belgium), and 3D spine model was established with threshold of 226-3071HU.

### Osteotomy simulation

3-CO and correction procedures were simulated in 3-Matic Medical 13.0 (Materialise NV, Leuven, Belgium) with cut tool and trim tool in 3D spine model. After trimming the targeted elements, the rotation tool was used to achieve the osteotomy gap closing procedure. The simulation of PSO and VCR were accomplished following the literature and surgeon’s experience. For patients with kyphoscoliosis, correction simulation was performed after sagittal procedure. The planning osteotomy angle was assessed according to the angle change of osteotomy gap in pre- and post-simulation 3D spine model.

### Osteotomy guide template design and manufacture

The osteotomy guide plate will consist of three parts, including the adjacent segment pedicle screw implantation guidance part, the lamina surface fitting part, and the osteotomy trajectory guidance part, which has been described in detail in our previous study [1]. Firstly, in Mimics Medical 21.0 (Materialise NV, Leuven, Belgium), the Lasso command was used to fill and repair the bone structure of the spinal model to build a repaired Mask for spine structure. Then, the Cylinder command was used to draw a cylindrical structure that mimics the pedicle screw implantation, and adjust the cylindrical trajectory structure to the optimal position for internal fixation through three-plane views of vertebrae, thereby completing the screw trajectory design of adjacent fixed segments. Import the spinal modification model and pedicle screw trajectory data into the 3-Matic Medical 13.0 (Materialise NV, Leuven, Belgium) software in STL format for osteotomy guide plate fabrication. Use a Wave Brush Mask to draw on the simulated pedicle screw cylindrical structure and nearby lamina surface (including osteotomy segments) to obtain personalized anatomical markers attached to the osteotomy and screw placement segments. Then use the Uniform Offset tool to deviate the contour area to create an attached guide plate model. Afterwards, the length of the osteotomy guide plate was measured using 3-Matic Medical 13.0 (Material NV, Leuven, Belgium), and the guide rail was drawn using ProE (Parameter Technology Corporation, Boston, MA, USA) software, which will serve as the osteotomy trajectory for the ultrasound osteotome. After these programs are completed, position calibration is performed using Mimics Medical 21.0 (Materialise NV, Leuven, Belgium) to ensure that the produced osteotomy guide rail overlaps with the osteotomy plane. Use the “Cut Orthogonal to Screen” command to cut the appropriate length and use the Boolean command to integrate it with the previous guide template. Finally, the computer-designed osteotomy guide plate template is 3D printed using resin as the printing material to create a solid guide plate template. The 3D spine model should also be 3D printed at the original 1:1 scale, and the 3D printed osteotomy guide template will be tested on the 3D printed spine model.

### Surgical procedure

**Fig. 1:**
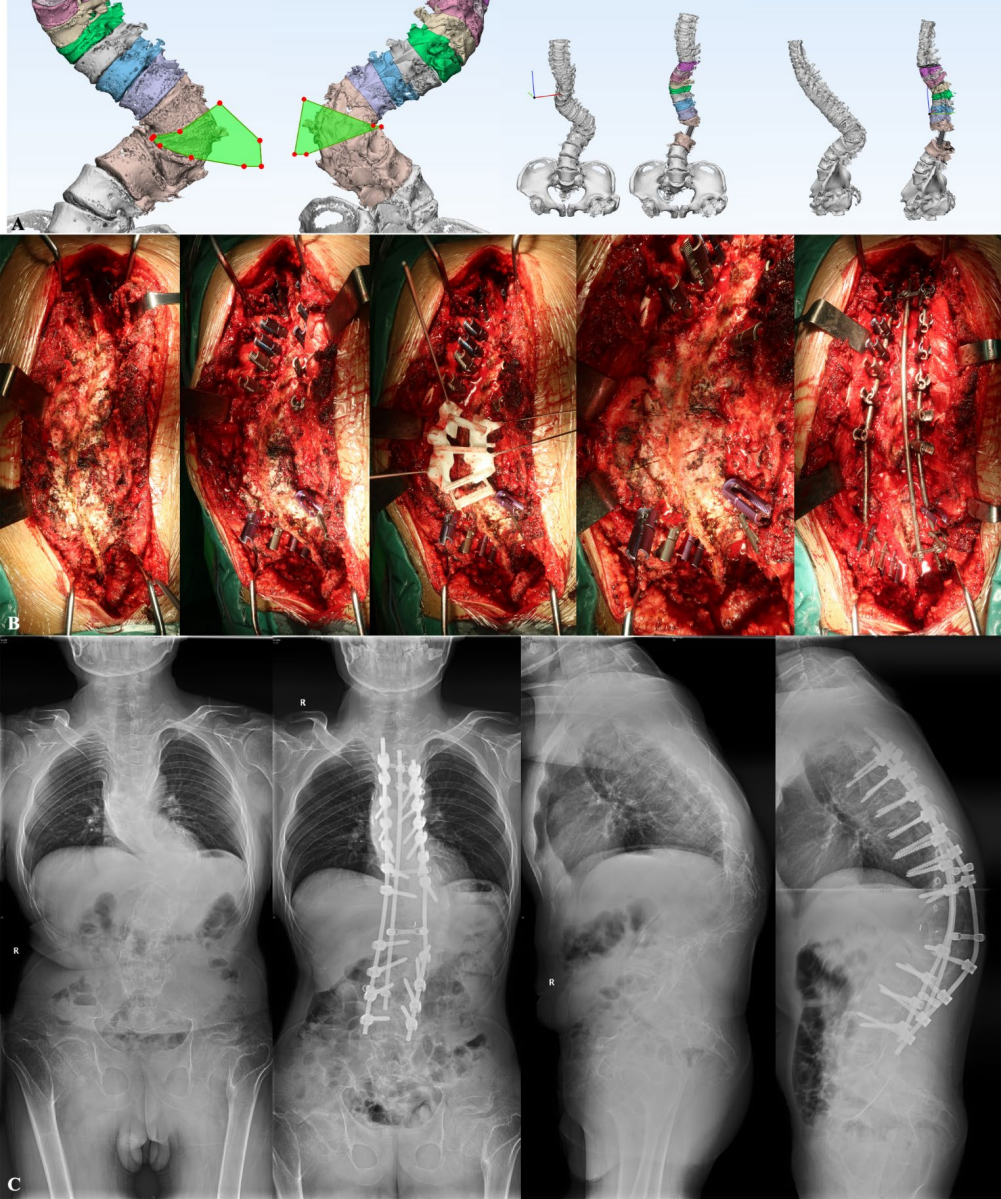
A 22-year-old male with congenital kyphoscoliosis undergoing three-column osteotomy at T12 and posterior spinal fusion surgery. (**A**) personalized computer-assisted three-dimensional simulation; (**B**) Execution of simulation through three dimensional-printed guide template; (**C**) The pre- and postoperative full-spine radiography

**Fig. 2:**
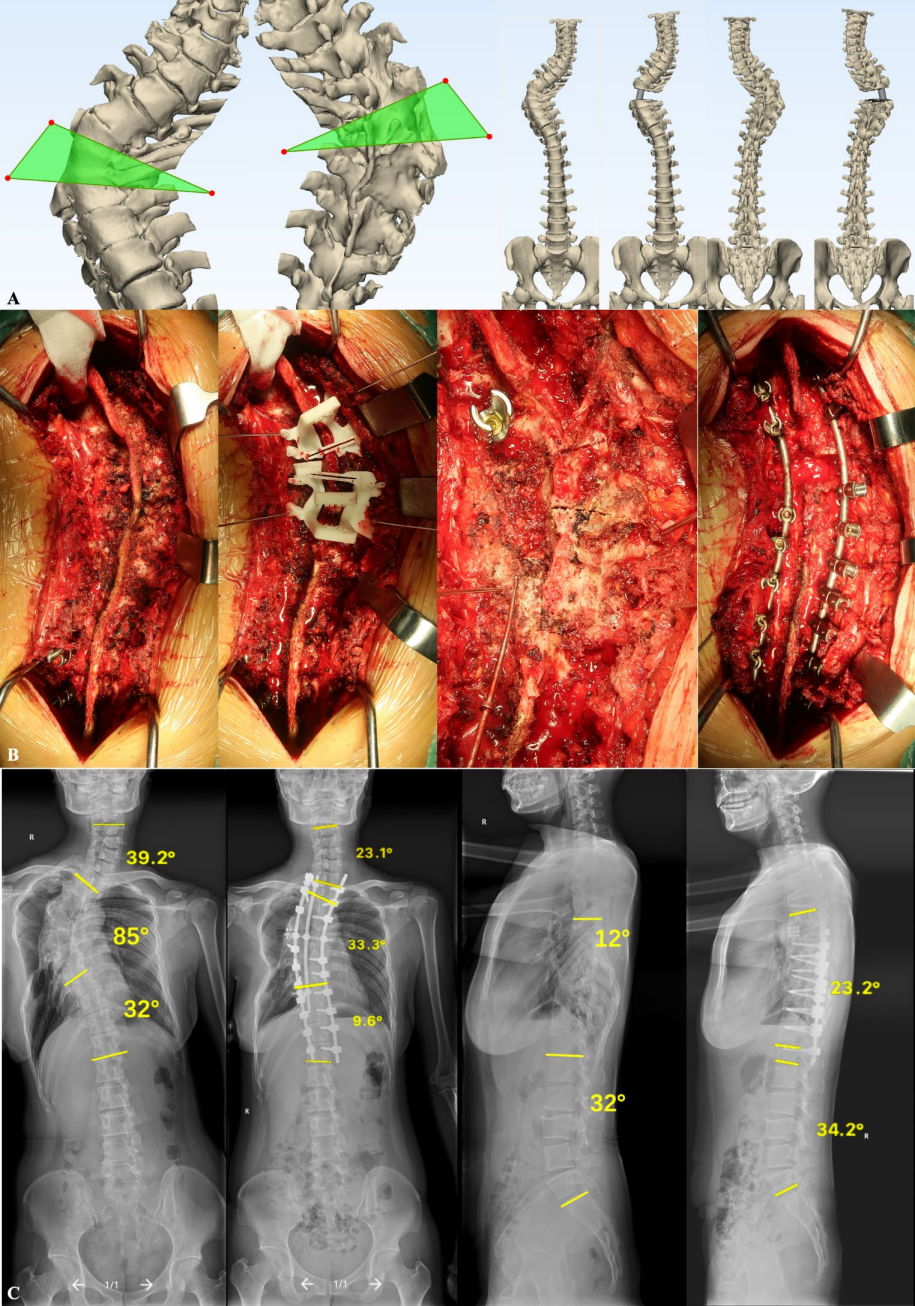
A 34-year-old female with congenital scoliosis undergoing three-column osteotomy at T5 and posterior spinal fusion surgery. (**A**) personalized computer-assisted three-dimensional simulation; (**B**) Execution of simulation through three dimensional-printed guide template; (**C**) The pre- and postoperative full-spine radiography

The pedicle screw trajectory template was placed firstly on the surface of the lamina and spinous process, ensuring firm bone contact. Specific anatomical landmarks could warrant the perfect match of the 3D-printed guide template with patients. The Kirschner wires were inserted into the pedicle through the foraminule in the guide template to fix the guide template. Then the ultrasonic osteotome was used to resect the lamina along the guide rail of the 3D-printed osteotomy guide template. Next, the 3D-printed guide template is removed and conventional PSO or VCR procedures were performed with a temporary rod. The osteotomy gap was gradually closed by repeated manual compression; however, overshortening of the spinal cord should be avoided. In the non-template group, the 3D spine model would be used to assist the osteotomy procedure. Two representative cases were demonstrated in Figs. 1 and 2.

### Data collection

The baseline clinical characteristics of the patients were collected, including age, gender, BMI, and diagnosis. The operating time (ORT), estimated blood loss (EBL), fusion levels, the location of 3CO, and the deviations as well as the match ratio of simulated and executed osteotomy angle in the coronal/sagittal plane were collected. Executed osteotomy angle was evaluated in the postoperative CT scan. Events of intraoperative neuromonitoring signal decline, postoperative neurological deficit, and postoperative non-neurological complications were recorded. Neurological deficit was defined as the decline of lower extremity motor score (LEMS) after surgery compared with the baseline LEMS. For radiographic parameters, the preoperative and postoperative main curve Cobb angle, FK, lumbar lordosis (LL), pelvic tilt (PT), sacral slope (SS), sagittal vertical axis (SVA), distance between C7 plumb line and centre sacral vertical line (C7PL-CSVL), and pelvic incidence (PI) were measured on the standing full-length spine radiographs. The health-related quality of life for patients in the template group was also recorded using Scoliosis Research Society-22r (SRS-22r).

### Statistical analysis

All statistical analyses were performed utilizing SPSS 24.0 (Chicago, IL, USA). To minimize the effects of confounding factors, the propensity score matching (PSM) was performed with a match tolerance of 0.01 according to the age and gender, based on previous literature [19, 20]. A 1:1 neighbour matching algorithm was applied with a calliper width of 0.2 in patients in the template group and non-template group. After matching, the absolute standardized mean difference (ASMD) was used to judge the balance of each covariate between groups. An ASMD ≤ 0.1 could be considered as balance between groups. The Shapiro-Wilk test was performed to determine whether continuous variables had a normal distribution. Continuous variables with a normal distribution are presented as the mean ± standard deviation; otherwise, the median and interquartile range are used. The counts and percentages are presented for categorical variables. For the comparison of continuous variables between groups, the independent-sample t-test or nonparametric Mann-Whitney U test was performed; for categorical variables, the Pearson chi-square test or Fisher’s exact test was applied. A two-sided P value of less than 0.05 was considered statistically significant.

## Results

A total of 40 patients (24 males and 16 females) with severe and complex ASD were retrospectively recruited in this study. The mean age at surgery was 36.53 ± 11.98 years. Among the patients, 20 were in the template group and 20 were in the non-template group. Based on ASMD, age and gender were adequately balanced (ASMD < 0.1). There were no significant differences in baseline characteristics and EBL between groups. However, the ORT in the template group was significantly shorter than the non-template group (359.25 ± 57.79 min vs. 398.90 ± 59.48 min, *P* = 0.039). The demographic characteristics and surgical information were presented in Table 1.

**Table 1:** Comparison of demographic characteristics and surgical information between the template and non-template groups

	Template group (n = 20)			Non-template group (n = 20)			P value
**Age**	36.70	±	12.44	36.25	±	11.83	0.769
**Gender**							1.000
Male	12			12			
Female	8			8			
**BMI**	23.72	±	1.98	23.62	±	1.94	0.864
**Etiology**							0.994
Post-tubercular	4 (20.0%)			5 (25.0%)			
Congenital	7 (35.0%)			7 (35.0%)			
Post-traumatic	1 (5.0%)			1 (5.0%)			
Ankylosing spondylitis	3 (15.0%)			3 (15.0%)			
Revision	5 (25.0%)			4 (20.0%)			
**Location of 3-CO**							0.833
Upper to T9	3 (15.0%)				3	(15.0%)	
T10 to L2	15 (75.0%)				16	(75.0%)	
Lower to L2	2 (10.0%)				1	(10.0%)	
**Fusion levels**	12.50	±	2.24	12.15	±	2.11	0.509
**Operating time**	359.25	±	57.59	398.90	±	59.48	0.039
**Estimated blood loss**	800.00	(500.00, 1150.00)		992.50	(662.50, 1410.00)		0.060
**Follow-up**	14.80	±	2.14	15.10	±	2.27	0.663

The preoperative FK was 92.72° ± 36.77° in the template group and 93.47° ± 33.91° in the non-template group, with a main curve Cobb angle of 63.35° (15.00°, 92.25°) and 64.00° (20.25°, 99.20°), respectively (Table 2). There were no significant differences in preoperative FK and main curve Cobb angle between the groups (*P* > 0.05). The correction effects were comparative in the two groups. Following the correction surgery, the postoperative FK was 41.96° ± 16.83° in the template group and 42.77°± 16.45° in the non-template group, with a main curve Cobb angle of 16.90° (1.25°, 40.75°) and 20.25° (5.00°, 50.60°), respectively. There were no significant differences in postoperative FK, postoperative main curve Cobb angle, correction rate of FK (54.20% vs. 51.94%, *P* = 0.738), and correction rate of main curve Cobb angle (72.41% vs. 61.33%, *P* = 0.101) between the groups. Also, there were no significant differences in PI, LL, PT, SS, SVA, and C7PL-CSVL between the groups (*P* > 0.05).

**Table 2:** Comparison of radiographic parameters between the template and non-template groups

	Template group (n = 20)			Non-template group (n = 20)			P value
**Focal kyphosis**							
Preoperative	92.72 ± 36.77			93.47 ± 33.91			0.904
Postoperative	41.96	±	16.83	42.77	±	16.45	0.878
Correction rate	54.20% ± 18.66%			51.94% (47.08%, 58.86%)			0.738
**Main curve Cobb angle**							
Preoperative	63.35 (15.00, 92.25)			64.00 (20.25, 99.20)			0.620
Postoperative	16.90 (1.25, 40.75)			20.25 (5.00, 50.60)			0.383
Correction rate	72.41%	±	23.06%	61.33%	±	18.33%	0.101
**Lumbar lordosis**							
Preoperative	51.54	±	27.43	50.05	±	20.74	0.847
Postoperative	50.20	±	15.12	50.89	±	9.88	0.864
**Pelvic tilt**							
Preoperative	19.90	±	15.05	20.83	±	11.48	0.874
Postoperative	13.88	±	4.56	16.14	±	3.67	0.092
**Sacral slope**							
Preoperative	31.15 (22.13, 34.35)			26.18 ± 11.00			0.758
Postoperative	31.67	±	9.57	28.63	±	8.59	0.300
**Sagittal vertical axis**							
Preoperative	35.25 (15.75, 80.33)			36.70 (25.70, 70.72)			0.883
Postoperative	25.22	±	18.60	27.77	±	14.43	0.630
**C7PL-CSVL**							
Preoperative	27.10 ± 15.61			26.10 (19.68, 38.78)			0.565
Postoperative	18.36	±	11.37	20.47	±	9.51	0.543
**Pelvic incidence**	44.85	±	10.09	46.25	±	9.36	0.652

The simulation, execution, and the deviations of osteotomy angle were presented in Table 3. There were no significant differences in simulated and executed osteotomy angle between the groups; however, in both coronal and sagittal planes, the execution-simulation deviations of osteotomy angle was significantly less in the template group than the non-template group coronal: 4.00° (1.20°, 5.50°) vs. 8.55°± 3.07°, *P* < 0.001; sagittal: 4.20 ± 2.10° vs. 9.10 ± 2.14°, *P* < 0.001, with a significantly greater match ratio of execution to simulation osteotomy angle (coronal: 89.90% vs. 74.50%, *P* < 0.001; sagittal: 90.45% vs. 80.35%, *P* < 0.001). There were no significant differences in precision of execution among patients with various aetiologies.

**Table 3:** Comparison of simulation, execution and deviation of osteotomy angle between the template and non-template groups

	Template group (n = 31)			Non-template group (n = 31)			P value
**Simulation**							
Coronal	29.12	±	14.97	31.02	±	14.15	0.657
Sagittal	45.12	±	15.58	46.85	±	15.35	0.759
**Execution**							
Coronal	26.30	±	13.39	25.90	±	14.96	0.930
Sagittal	42.53	±	13.87	40.66	±	18.05	0.544
**Deviation**							
Coronal	4.00 (1.20, 5.50)			8.55	±	3.07	< 0.001
Sagittal	4.20	±	2.10	9.10	±	2.14	< 0.001
**Match ratio**							
Coronal	89.90%	±	6.43%	74.50% (64.00%, 78.75%)			< 0.001
Sagittal	90.45%	±	4.16%	80.35% (76.25%, 83.00%)			< 0.001

Intraoperative neuromonitoring signal decline was detected in one and four patients in the template group and non-template group, respectively (Table 4). The incidence of intraoperative neuromonitoring signal decline events was lower in the template group than the non-template group (5.0% vs. 20.0%), although it was not statistically significant (*P* = 0.152). Postoperative neurological deficit was observed in one patients in the templates group and seven patients in the non-template group. The incidence of postoperative neurological deficit was significantly lower in the template group than the non-template group (5.0% vs. 35.0%, *P* = 0.018). The LEMS of the patient with postoperative neurological deficit in the template group recovered to the baseline level at the final follow-up; however, the declined LEMS of one patient in the non-template group still remained.

**Table 4:** Comparison of complications between the template and non-template groups

	Template group (n = 20)	Non-template group (n = 20)	P value
**IONM signal decline**	1 (5.0%)	4 (20.0%)	0.152
**Postoperative neurological deficit**	1 (5.0%)	7 (35.0%)	0.018*
Recovered at the final FU	1 (5.0%)	6 (30.0%)	0.038*
Unrecovered at the final FU	0 (0.0%)	1 (5.0%)	0.311
**Non-neurological complications**	3 (15.0%)	8 (40.0%)	0.077
Surgical site infections	1 (5.0%)	2 (10.0%)	0.548
Hematoma	0 (0.0%)	1 (5.0%)	0.311
Pneumothorax	0 (0.0%)	1 (5.0%)	0.311
Dural tears	1 (5.0%)	2 (10.0%)	0.548
Pneumonia	1 (5.0%)	2 (10.0%)	0.548

IONM, intraoperative neurophysiological monitoring  * Bolded P values indicate statistically significant difference.

Eleven postoperative non-neurological complications were recorded in 10 patients (Table 4). The incidence of postoperative non-neurological complication was slightly lower in the template group than the non-template group (15.0% vs. 40.0%), although it was not statistically significant (*P* = 0.077). There were no significant difference in the incidence of surgical site infection (5.0% vs. 10.0%, *P* = 0.548), dural tears (5.0% vs. 10.0%, *P* = 0.548), hematoma (0.0% vs. 5.0%, *P* = 0.311), pneumothorax (0.0% vs. 5.0%, *P* = 0.311), and pneumonia (5.0% vs. 10.0%, *P* = 0.548). All the patients with surgical site infection underwent intravenous antibiotic treatment. One patient with hematoma in the non-template group underwent reoperation immediately after postoperative physical examination and CT scan. By paired t-test, there were significant differences in domains of mental health (3.96 ± 0.24 vs. 4.33 ± 2.53, *P* < 0.001), self-image (2.86 ± 0.26 vs. 3.76 ± 0.27, *P* < 0.001), function (3.56 ± 0.43 vs. 3.83 ± 0.43, *P* < 0.001), and total SRS-22r scores (3.63 ± 0.22 vs. 4.02 ± 0.21, *P* < 0.001) at the final follow-up in the template group.

## Discussion

The surgical treatment of severe and complex ASD required skilled technique and experience due to the abnormal anatomy of the spine, such as congenital deformity or history of spinal fusion [1]. For such cases, not only the severity but also the rigidity of spinal deformity made the radical 3-CO mandatory. As the osteotomy site was commonly around the apex of the spinal deformity, adjacent to the spinal cord, the risk of postoperative neurologic deficit was high [21].

Also, the difficulties in accurate identifying anatomical landmarks hindered surgeons to follow the surgical planning. Therefore, performing 3-CO effectively and safely is of great importance to a successful deformity correction surgery. In our previous study, we applied personalized computer-assisted three-dimensional simulation for patients with thoracolumbar kyphosis secondary to AS requiring 3-CO; however, the actual closure of osteotomy gap could not be matched exactly with the planning and a desired osteotomy could not be achieved [9]. Therefore, determining how to precisely translate the virtual planning to practice was still challenging. Currently, an increasing number of surgeons had tried to execute personalized surgical planning with assistance of 3D printing [22, 23]. Unlike CT navigation, which requires intraoperative scanning, or robotic system, which requires time for setup and sometimes intraoperative adjustments, guide templates could potentially reduce the overall operating time and exposure to radiation. Once in place, the template guide the necessary operation without additional imaging or adjustment. Also, using guide template could be as less technologically complex and more cost-effective compared to robotic system or advanced intraoperative CT navigation. In addition, the learning curve for using guide templates might be considered less steep. Surgeons who familiar with conventional surgical approaches may find integrating guide templates into their practice more straightforward. For spinal deformity surgery, individualized 3D-printed pedicle screw guide template has been used, achieving a higher accuracy of insertion than free-hand technique [15]. Although a few studies have reported the feasibility of osteotomy template guide in severe and complex ASD, to the best of our knowledge, this was first study to investigate the precision, efficacy, and safety of 3D-printed osteotomy guide template through comparative matched-cohort study.

Due to the severe scoliosis or kyphotic deformity, the tension of the spinal cord in the apical region was high and the blood flow was impacted [24]. Therefore, an improper closure of osteotomy gap could lead to postoperative neurological complications. Iyer et al. reported that the incidence of neurological complication was as high as 13.3% in patients with rigid spinal deformity requiring 3-CO [25]. The Scoli-RISK-1 study presented that the percentage of patients who had a LEMS decline after undergoing complex spinal reconstructive surgery for ASD was 23.0% at discharge [7]. In the current study, we found that the incidence of postoperative neurological deficits was significantly decreased with the application of 3D-printed guide template for osteotomy (5.0% vs. 35.0%). We considered that the 3D-printed guide template could assist surgeons to identify the designed location of the osteotomy through the complex spinal structures and made the designed osteotomy gap to close, which was correlated with shortening of spinal cord.

Determining the precision of the surgical technique is crucial before the wide clinical application, particularly in surgery for severe and complex ASD. In the current study, the postoperative evaluation by CT scan revealed a significantly higher precision of osteotomy execution using 3D-printed osteotomy guide template. The executed osteotomy angle in both coronal (89.90% vs. 74.50%) and sagittal plane (90.45% vs. 80.35%) was more consistent with the preoperative simulation, compared with the non-template group. Although we tried to completely close the osteotomy gap, the overshortening of spinal cord inhibited this procedure. We considered that this was the most important factor impacting the precision of execution. Additionally, in contrast to free-hand osteotomy, in which the laminae are usually addressed gradually, the major advantage of 3D-printed guide template was allowing surgeons to directly and effectively perform the osteotomy using ultrasonic osteotome, which would decrease the disturbance to spinal cord and shorthorn the ORT. For patients with unique spinal posterior anatomical structures, like congenital, iatrogenic or tubercular ASD, this effect would be more obvious.

The findings of our study highlight the transformative potential of incorporating 3D-printed osteotomy guide templates in 3-CO procedures for severe and complex ASD. Beyond enhancing precision and reducing the risk of postoperative neurological deficits, our research indicates that this innovative approach could mitigate the demands on surgeons’ experience and skills. The structured and guided nature of 3D-printed templates offers a standardized pathway, diminishing the reliance on intricate surgical expertise. This aspect not only ensures a more consistent execution of the procedure but also holds the promise of democratizing access to 3-CO procedures, and more clinical centres could undertake the complex procedures for severe and complex ASD cases. Our study encourages further exploration and collaboration within the healthcare settings to validate and expand upon these findings, with the ultimate goal of informing evidence-based guidelines and fostering a more standardized and accessible approach in the field of spinal surgery.

Although the various advantages of personalized 3D-printed osteotomy guide template, such as 3D insight of the case-specific anatomy, identification of osteotomy location, and translation of the surgical planning or simulation to the real surgical site, the application was still limited to the first stages of 3CO, such as laminectomy [26]. The two reasons were that the 3D-printed guide template could not be placed simultaneously with temporary rods and it could not effectively guide the osteotomy trajectory in the anterior and middle columns. In the future, optimized inlets for rod positioning should be designed. A combination of guide template and augmented-reality navigation may be a better alternative, which warrants further scientific investigations [27].

### Limitation

Several limitations should be noted in this study. First, the limited number of cases in the present study constitutes a potential limitation, and only two of the most important confounding factors (age and gender) were selected for PSM. To substantiate the merits of personalized 3D-printed guide template for osteotomy, a multicenter comparative study should be conducted. Another limitation was the retrospective study design. Although propensity-matched method was used, more advanced study designs, such as randomized controlled trials, should be considered in the future studies to enhance the robustness of findings. We did have plans for a follow-up study to assess the long-term outcomes of the 3D-printed guide template treatment. In the long-term follow up duration, we would focus on the correction maintenance, instrumentation-related complication, bony fusion status, and activities of daily living.

## Conclusion

Performing 3-CO with the assistance of personalized 3D-printed guide template could increase the precision of execution, decrease the risk of postoperative neurological deficit, and shorten the ORT in the correction surgery for severe and complex ASD. The personalized osteotomy guide had the advantages of 3D insight of the case-specific anatomy, identification of osteotomy location, and translation of the surgical planning or simulation to the real surgical site.

## Data Availability

No datasets were generated or analysed during the current study.
